# Study on ZrB_2_-Based Ceramics Reinforced with SiC Fibers or Whiskers Machined by Micro-Electrical Discharge Machining

**DOI:** 10.3390/mi11110959

**Published:** 2020-10-26

**Authors:** Mariangela Quarto, Giuliano Bissacco, Gianluca D’Urso

**Affiliations:** 1 Department of Management, Information and Production Engineering, University of Bergamo, Via Pasubio 7/b, 24044 Dalmine, Italy; gianluca.d-urso@unibg.it; 2 Department of Mechanical Engineering, Technical University of Denmark, Produktionstorvet, Building 425, 2800 Kgs. Lyngby, Denmark; gibi@mek.dtu.dk

**Keywords:** micro-EDM, Zirconium Boride, silicon carbide fibers, silicon carbide whiskers, advanced material

## Abstract

The effects of different reinforcement shapes on stability and repeatability of micro electrical discharge machining were experimentally investigated for ultra-high-temperature ceramics based on zirconium diboride (ZrB_2_) doped by SiC. Two reinforcement shapes, namely SiC short fibers and SiC whiskers were selected in accordance with their potential effects on mechanical properties and oxidation performance. Specific sets of process parameters were defined minimizing the short circuits in order to identify the best combination for different pulse types. The obtained results were then correlated with the energy per single discharge and the discharges occurred for all the combinations of material and pulse type. The pulse characterization was performed by recording pulses data by means of an oscilloscope, while the surface characteristics were defined by a 3D reconstruction. The results indicated how reinforcement shapes affect the energy efficiency of the process and change the surface aspect.

## 1. Introduction

Among the advanced ceramic materials, ultra-high-temperature ceramics (UHTCs) are characterized by excellent performances in extreme environments. This family of materials is based on borides (ZrB_2_, HfB_2_), carbides (ZrC, HfC, TaC), and nitrides (HfN), which are marked by high melting point, high hardness and good resistance to oxidation. In particular, ZrB_2_-based materials are of particular interest because of their suitable properties combination and are considered attractive in applications, such as a component for the re-entry vehicles and devices [1,2,3,4].

The relative density of the base material ZrB_2_ is usually about 85% because of the high level of porosity of the structure, furthermore, in the last years, researchers are focused on fabricating high-density composites characterized by good strength (500–1000 MPa). For these reasons, the use of single-phase materials is not sufficient for high-temperature structural applications. Many efforts have been done on ZrB_2_-based composites in order to improve the mechanical properties, oxidation performances, and fracture toughness; however, the low fracture toughness remains one of the greatest limitations for the application of these materials under severe conditions [5,6,7,8,9,10]. Usually, the fracture toughness of ceramic materials can be improved by incorporating appropriate additives that activated toughening mechanisms such as phase transformation, crack pinning, and deflection. An example is the addition of SiC, in fact, it has been widely proved that its addition improves the fracture strength and the oxidation resistance of ZrB_2_-based materials due to the grain refinement and the formation of a protective silica-based layer. Based on these aspects, recent works have focused on ZrB_2_-based composites behavior generated by the addition of SiC with different shapes (e.g., whiskers or fibers). Specifically, it has been reported that the addition of whiskers or fibers gives promising results, improving the fracture toughness and this improvement could be justified by crack deflection [5,6,9,11,12]. The critical aspect of the reinforced process was the reaction or the degeneration of the reinforcement during the sintering process [13,14].

Despite all the studies that aim to improve mechanical properties and resistance, this group of materials is very difficult to machine by the traditional technologies, because of their high hardness and fragility. Only two groups of processes are effective in processing them: On one side the abrasive processes as grinding, ultrasonic machining, and waterjet, on the other side the thermal processes as laser and electrical discharges machining (EDM) [15,16,17,18].

In this work, ZrB_2_ materials containing 20% vol. SiC whiskers or fibers produced by hot pressing were machined by the micro-EDM process; in particular, it would investigate the effect of the non-reactive additive shapes on the process performances, verifying if the process is stable and repeatable for advanced ceramics, and in particular for materials characterized by different geometry of the additive. The choice of 20% vol. is related to the evidence reported in some previous works [14,18,19], in which it has been shown that this fraction of additive has allowed generating the best combination of oxidation resistance and mechanical characteristics useful for obtaining better results in terms of process performances and dimensional accuracy for features machined by micro-EDM.

## 2. Materials and Methods

The following ZrB_2_-based composites, provided by ISTEC-CNR of Faenza (Consiglio Nazionale delle Ricerche—Istituto di Scienza e Tecnologia dei Materiali Ceramici, Faenza (RA)—Italy), have been selected for evaluating the influence of additive shape on the process performances and geometrical of micro-slots machined by micro-EDM technology:

- ZrB_2_ + 20% SiC short fibers, labelled as ZrB20f
- ZrB_2_ + 20% SiC whiskers, labelled ZrB20w.

Such as reported in [14], commercial powders were used to prepare the ceramic composites: ZrB_2_ Grade B (H.C. Starck, Goslar, Germany), SiC HI Nicalon-chopped short fibers, Si:C:O = 62:37:0.5, characterized by 15 μm diameter and 300 μm length or SiC whiskers characterized by average diameter 1 μm and average length 30 μm.

The powder mixtures were ball milled for 24 h in pure ethanol using silicon carbide media. Subsequently, the slurries have been dried in a rotary evaporator. Hot-pressing cycles were conducted in low vacuum (100 Pa) using an induction-heated graphite die with a uniaxial pressure of 30 MPa during the heating and were increased up to 50 MPa at 1700 °C (T_MAX_), for the material containing fibers, and at 1650 °C (T_MAX_) for the composites with whiskers. The maximum sintering temperature was set based on the shrinkage curve. Free cooling followed. Details about the sintering runs are reported in Table 1, where T_ON_ identify the temperature at which the shrinkage started. Density was estimated by the Archimedes method.

**Figure 1:**
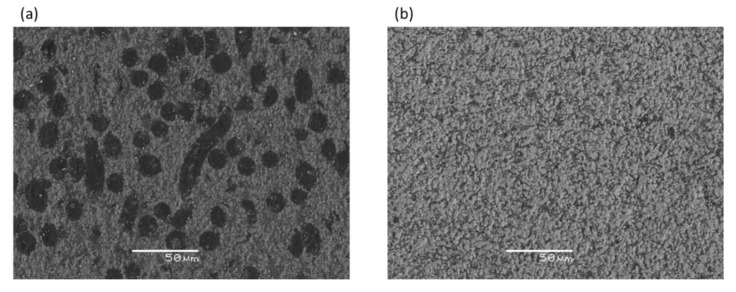
SEM backscattered images of typical appearance of ZrB20f (**a**) and ZrB20w (**b**).

The raw materials were analyzed by Scanning Electron Microscopy (SEM) (Figure 1). The samples reinforced by the fibers show a very clear separation between the base matrix and the non-reactive additive. Fibers dispersion into the matrix was homogenous, as no agglomeration was observed in the sintered body. However, some porosity was retained in the microstructure. The fibers showed a tendency to align their long-axis perpendicular to the direction of applied pressure. It is apparent that their length was significantly reduced compared with the starting dimensions, as the maximum observed length was about 300 µm. For the sample containing whiskers reinforcement, a dense microstructure was observed and the whiskers are generally well dispersed into the matrix. The mean grain size of ZrB_2_ grains was slightly lower (2.1 ± 0.2 µm) than the base material (3.0 ± 0.5 µm); furthermore, the whiskers showed a tendency to form large bundles. The addition of SiC whiskers promoted both strengthening and toughening compared with the base material ZrB_2_. The maximum toughness increase (5.7 MPa·m^1/2^) was of the order of 50% when whiskers were added. In the sample containing fibers, the increase in fracture toughness (5.5 MPa·m^1/2^) corresponds to a strength reduction. A direct observation of the crack morphology and the comparison with theoretical models demonstrates that, besides a residual stress contribution, the toughness increase was almost entirely explained in terms of crack deflection in the whiskers and in terms of crack bowing in the fibers. No crack bridging was obtained as the reinforcement pullout was hindered by the formation of interphases or intergranular wetting phases, which promoted a strong bonding between matrix and reinforcement. The values of fracture toughness of the ZrB20f and ZrB20w were close to those of hot-pressed composites previously. This indicates that, even if a more efficient thermal treatment is used, the nature of the matrix/reinforcement interface did not change notably [2].

## 3. Experimental Section

### 3.1. Experimental Set-Up

A simple circular pocket having a diameter equal to 1 mm and a depth of about 200 µm was selected as the test feature for machining experiments. These micro-features had been processed on the SARIX^®^ SX-200 machine (Sarix, Sant’Antonino, Switzerland) by micro-EDM milling. Solid tungsten carbide electrode with a diameter equal to 300 µm was used as a tool; while the dielectric fluid was formed by hydrocarbon oil. The experiments were performed for three different process parameters settings, corresponding to different pulse shapes. It is essential to remark that the machine used for the experimental tests expresses some process parameters as indexes (e.g., peak current, width). The instantaneous values cannot be set, because the machine presents an autoregulating system. Thus, the characterization of electrical discharges population is very important not only to assess the real value of process parameters but, most of all, to evaluate the stability and repeatability of the process. Due to this characteristic of the machine, an acquisition system was developed to cover the gap. In particular, a current monitor and a voltage probe were connected to the EDM machine and to a programmable counter and a digital oscilloscope. These connections allow to acquire the current waveforms and count the discharges occurred during the process. Specifically, a current monitor with a bandwidth of 200 MHz and a Rohde & Schwarz RTO1014 oscilloscope were used. The counter has been set once the trigger value was established to avoid recording and counting the background noise.

Preliminary tests were performed to define the optimal process parameters for each combination of material and pulse type. The experimental campaign was based on a general full factorial design, featured by two factors: The additive shape, defined by two levels, and the pulse type, defined by three levels. Different levels of pulse types identify the different duration of the discharges, in particular, level A is referred to long pulses while level C identify the short pulses. Three repetitions were performed for each run.

### 3.2. Discharges Population Characterization

For each pulse types, combined with both materials, discharge populations have been characterized by repeated waveform samples of current and voltage signals. The current and voltage probes were connected to the digital oscilloscope having a real-time sample rate of 40 MSa/s. The trigger level of the current signal was set to 0.5 A in order to acquire all the effective discharges. The acquired waveform samples were stored in the oscilloscope buffer and then transferred to a computer to be processed by a Matlab code, written by the authors. The Matlab code was used to evaluate the numbers of electrical discharge, the current peak values, the duration (width), the voltage and to estimate the energy content in each discharge. Finally, the average value of energy per discharge (*E*) was estimated by integrating the instantaneous value of the power, calculated as the product of the instantaneous values of current (*i*(*t*)) and voltage (*v*(*t*)), with respect to the time (Equation (1)).

(1) $$E=\int_{0}^{T}v\left(t\right)\;\cdot \;i\left(t\right)dt$$

**Figure 2:**
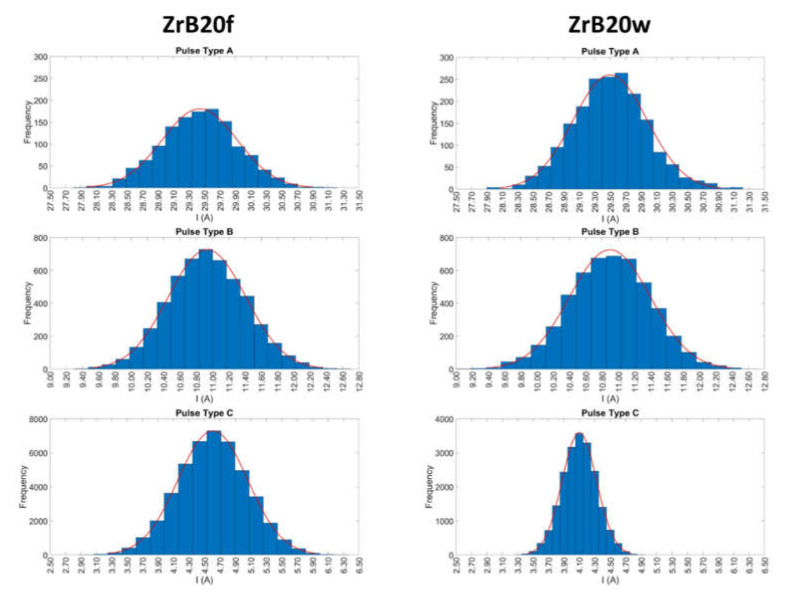
Examples of frequency distribution histograms for pulses occurred during ZrB20 machining.

To show the discharge population distribution for all pulse types and for both additive shapes, the peak current distribution histograms are plotted, showing the number of observed discharges, with peak current within discrete intervals, for all the (Figure 2). Histograms show good reproducibility and stability of the process, providing information regarding the frequency waveforms with different peaks of current. The discharge samples are well described by a normal distribution, which is characterized by a good reproducibility suggesting a stable process. Considering both additive shapes, the values of peak current are included in a similar range for pulse type A and B, while, for pulse type C can be identified a great difference; in fact, for whiskers, the range of variation of current peaks is smaller than for short fibers.

Thanks to the discharge characterization it was possible to define the real value of the process parameters. The pulse characteristics for the parameters setting estimated by the data elaboration with Matlab are reported in Table 2.

In terms of peak current and voltage, the differences between the two materials, machined by the same pulse type, were really tiny. This suggested that EDM machining on ZrB_2_-based composites reinforced by whiskers would have been characterized by higher machining speed.

### 3.3. Characterization Procedure

A 3D reconstruction of micro-slots was performed by means of a confocal laser scanning microscope (Olympus LEXT, Southend, Essex, UK) with a magnification of 20×. This microscope recognizes the peaks of the reflected light intensities of multiple layers and, setting each layer as the focal point, makes it possible to analyze and measure each layer. Then, the images were analyzed with an image processor software (SPIP 6.7.3, Image Metrology, Lyngby, Denmark), firstly performing a plane correction on all the images to level the surfaces and to remove primary profiles, then the surface roughness (Sa) was assessed, on the base of the international standard UNI EN ISO 25178:2017 by the real-topography method. The process performances were evaluated through the estimation of three indicators: The material removal per discharge (MRD), the tool wear per discharge (TWD), and the tool wear ratio (TWR).

MRD (Equation (2)) was calculated as the ratio of material removed from the workpiece (MRW (mm^3^)), estimated through SPIP, and the number of discharges (N) recorded by the programmable counter.

(2) $$\mathrm{MRD}=\frac{\mathrm{MRW}}{N}$$

Since this kind of materials is characterized by a high level of porosity, to get the actual values of MRD, the volume of the micro-slots was adjusted considering the relative density (δ—Table 1). for compensating the presence of porosity in the sample structure. The MRD is calculated as reported in Equation (3).

(3) $${\mathrm{MRD}}_{\delta }=\frac{\mathrm{MRW}\cdot \delta }{N}=\mathrm{MRD}\cdot \delta$$

TWD (Equation (4)) was estimated as the ratio between the material removed from the tool electrode (MRT (mm^3^)) and the number of discharges [N] recorded by the programmable counter. Tool wear was measured as the difference between the length of the electrode before and after the single milling machining. The length was measured through a touching procedure executed in a reference position. The electrode wear volume was estimated starting from the length of the tool wear and considering the tool such as a cylindrical part.

(4) $$\mathrm{TWD}=\frac{\mathrm{MRT}}{N}$$

Tool Wear Ratio (Equation (5)) was calculated as the ratio between the previous performances’ indicators considering the relative density of the workpiece material (TWR_δ_).

(5) $${\mathrm{TWR}}_{\delta }=\frac{\mathrm{TWD}}{\mathrm{MRD}}=\frac{\mathrm{MRT}}{\mathrm{MRW}\cdot \delta }$$

## 4. Results and Discussion

**Figure 3:**
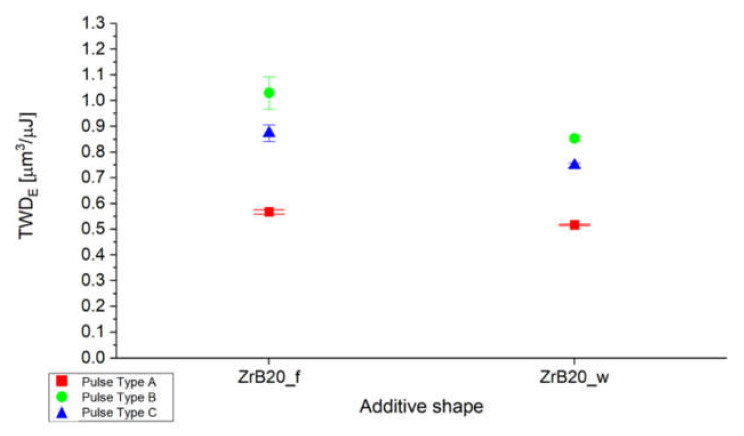
Average ratio between tool wear per discharge (TWD) and energy per discharge as a function of the additive shape and pulse type.

During the analysis, the energy efficiency of a single discharge was evaluated. Figure 3 shows the tool wear per discharge divided by the energy of single discharge (TWD_E_), as a function of the additive fraction and the pulse type. The plot shows a lower energy efficiency for pulse type A and this is a positive aspect because it indicates less impact on the electrode wear. For both additive shapes, the medium pulses are characterized by a higher impact on the tool wear.

**Figure 4:**
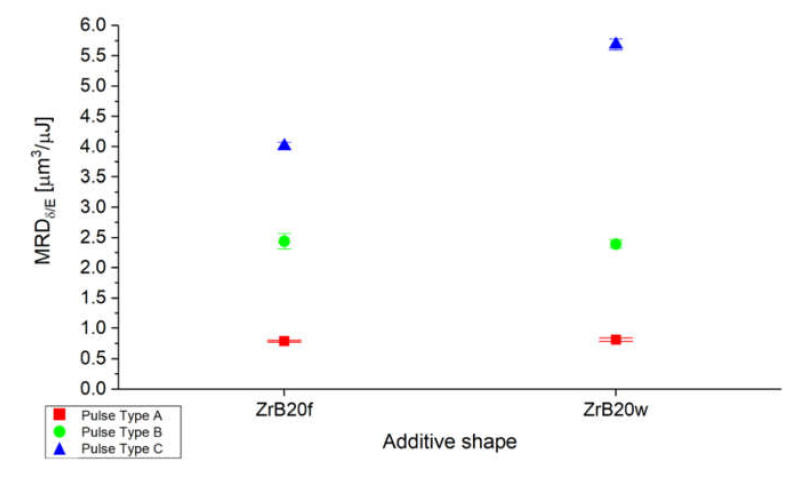
Average ratio between material removal per discharge (MRD) and energy per discharge estimated considering the relative density of the samples as a function of the additive shape and pulse type.

To evaluate the energy efficiency from the material removed point of view, as has already been done in the previous plot, also the material removal per single discharge was evaluate as the ratio with the energy for single discharge. In this case, the best results were obtained for pulse type C where a single discharge is characterized by lower duration and energy but and higher efficiency, such as illustrated in Figure 4.

**Figure 5:**
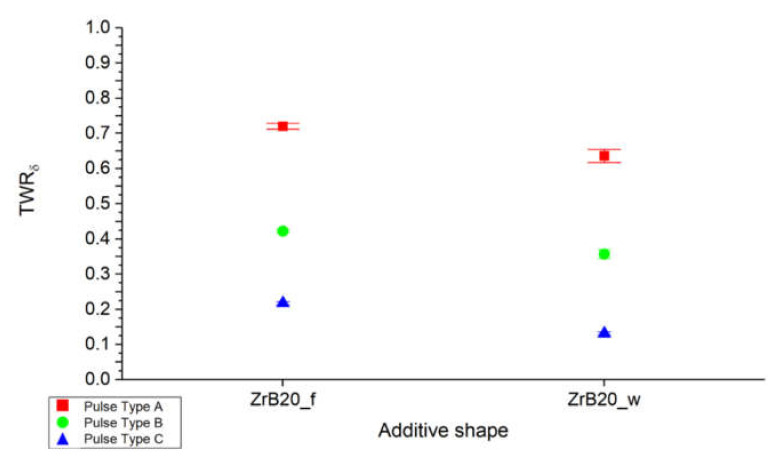
Average tool wear ratio (TWR) as a function of the additive shape and pulse type.

Figure 5 shows that the TWR for all pulse type is lower for specimens containing whiskers reinforcement. In particular, the whiskers allow to reduce the tool wear and increasing the material removal rate efficiency probably thanks to the dense microstructure and the reduction of the grains size.

In general, for pulse type A the energy per discharge is higher in comparison to others pulse types but for both Energy efficiency of TWD (TWD_E_) and Energy efficiency of MRD_δ_ (MRD_δ/E_), the energy efficiency is lower (positive aspect from TWD_E_ point of view). This can suggest a greater energy dispersion in machining performed with pulse type A than for others; in fact, energy per discharge of pulse type C is 40–50 times smaller than pulse type A but the energy efficiency is higher.

The factorial design was analyzed in order to comprehend which factors and interactions are statistically significant for the performance indicators and surface roughness. Table 3 shows the average results obtained from the experiments reporting the average and the standard deviation of MRD_δ_, TWD, TWR_δ_, and Sa for each level of the experimental design. A general linear model was used to perform a univariate analysis of variance, including all the main factors and their interactions.

The Analysis of Variance (ANOVA) results of the experimental plan are reported in Table 4 omitting the values related to the pulse type, since all the *p*-values results to be very close to 0.00. The parameters are statistically significant for the process when the p-value is less than 0.05 since a confidence interval of 95% is applied. As a general remark, all the indicators resulted to be influenced by the additive shape and the pulse type. In some cases (for MRD_δ_ and TWD), also the interaction showed an effect in terms of ANOVA. This aspect suggested that the interaction of factors is relevant for indicators that, in some way, can be correlated to the machining duration.

**Figure 6:**
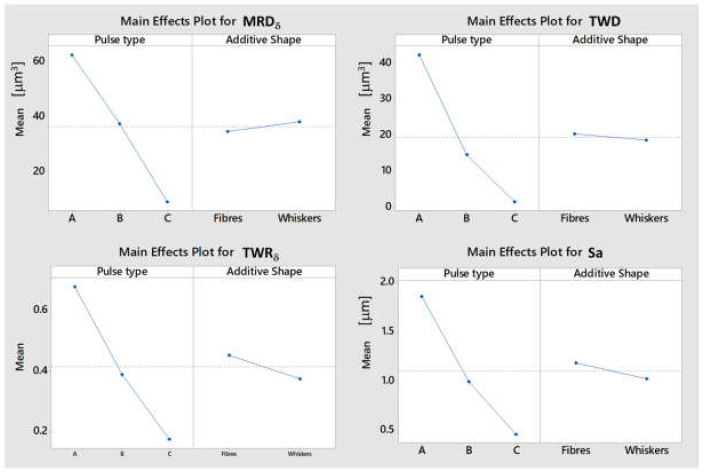
Main effects plot for indicators affected by pulse type and additive shape.

**Figure 7:**
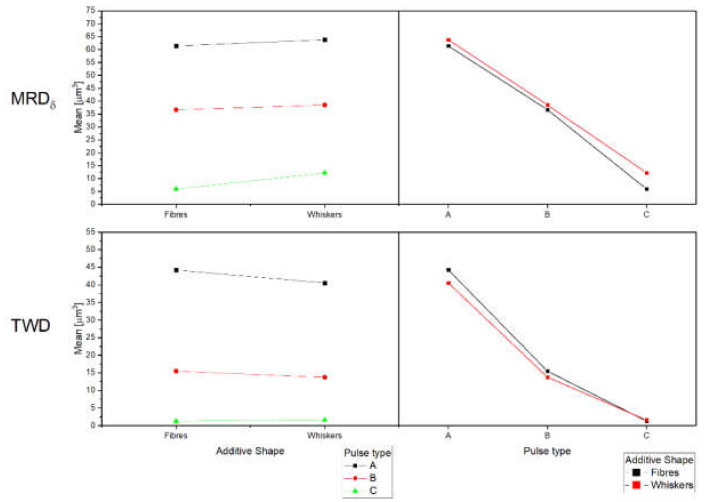
Interaction plot for MRD_δ_ and TWD.

Main effects plots (Figure 6) show that indicators are mainly influenced by the pulse type that establishes the range in which process parameters can vary, and in particular, the characteristics of the pulses. For all indicators, reduction in pulse duration and in peak current intensity generate the lower value of MRD_δ_, TWD, and TWR_δ_. At the same time, tests with whiskers additive generate increment in MRD_δ_ and in surface roughness, and a reduction in TWD and TWR_δ_. By increasing the pulse duration and peak current intensity from type C to type A, the machining speed (MRD_δ_) is 10 times greater, but the surface quality decreases by −60%. For MRD_δ_ and TWD, also the interaction between pulse type and additive shape influence the results. The interaction plots (Figure 7) give more information than the *p*-value showing that the weight of the interaction is slight. What it is possible to notice is that for the samples that are machined by pulse type C, the effect on MRD_δ_ is more evident, while from the TWD point of view it is more evident for pulse type A). In general, tests performed on materials with whiskers additive are characterized by better results, both in terms of process performances and surface finishing. In fact, optimal performance for ED-machining are characterized by a higher level of MRD_δ_, to perform a fast machining, and lower TWD, to reduce the waste of material related to the tool wear as a function of material removed from the workpiece.

**Figure 8:**
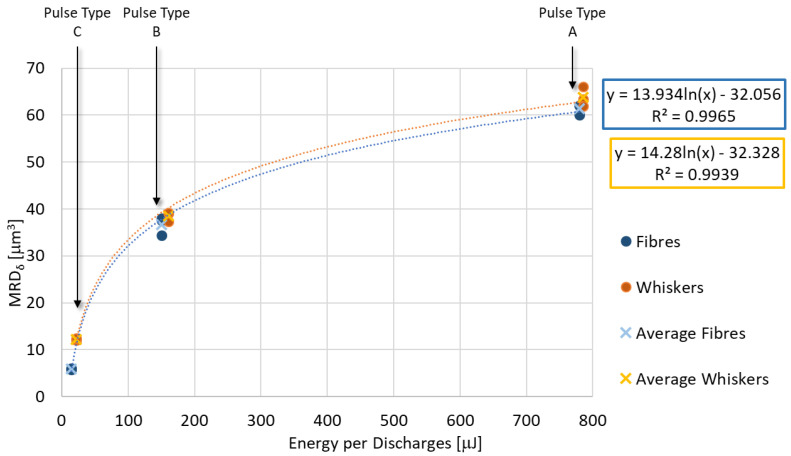
Material removal per discharge as a function of energy per discharge for both additive shapes and their regression equation.

**Figure 9:**
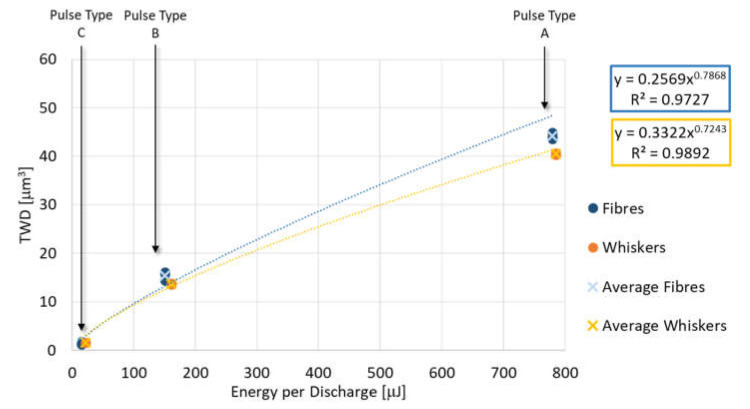
Tool wear per discharge as a function of energy per discharge for both additive shapes and their regression equation.

**Figure 10:**
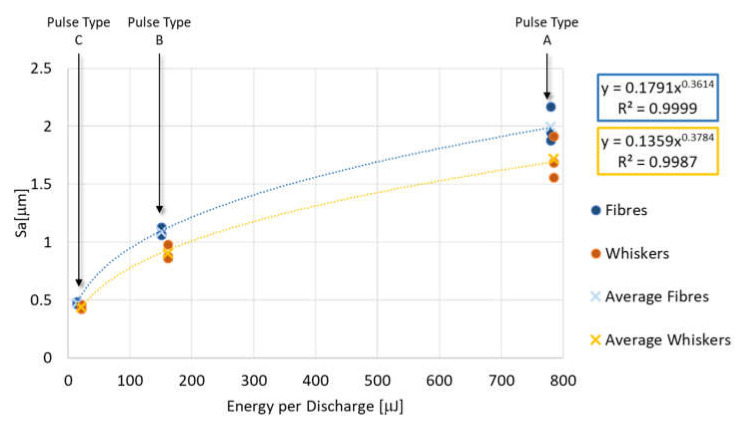
Surface roughness as a function of energy per discharge for both additive shapes and their regression equation.

Figure 8, Figure 9 and Figure 10 represent each indicator as a function of the energy per discharge and, regardless of the additive shape, it is possible to observe that each indicator is well represented (R^2^ ≈ 1) by the same type of regression equation for both additive shapes. TWD shows lower values of R^2^. Specifically, the MRD_δ_ is well described by a logarithmic regression equation (Equation (6) and Equation (7)), while the TWD and the Sa are well described by a power regression equation (Equations (8)–(11)). In all regression equation reported in the plots, y-axis is referred to the indicator, while x-axis is referred to the energy generated by a single discharge (E). For MRD_δ_ and TWD the differences as a function of additive shape are very small, in fact, despite they cover different ranges, the values are very close to each other. The situation is different for Sa, which is characterized by completely different ranges without overlap.

(6) $$\begin{array}{cccc} {\mathrm{MRD}}_{\delta \_f}=13.934\ln\left(E\right)-32.056 & & & R^{2}=0.9965 \end{array}$$

(7) $$\begin{array}{cccc} {\mathrm{MRD}}_{\delta \_w}=14.28\ln\left(E\right)-32.328 & & & R^{2}=0.9939 \end{array}$$

(8) $$\begin{array}{cccc} {\mathrm{TWD}}_{f}=0.2569E^{0.7868} & & & R^{2}=0.9727 \end{array}$$

(9) $$\begin{array}{cccc} {\mathrm{TWD}}_{w}=0.3322E^{0.7243} & & & R^{2}=0.9892 \end{array}$$

(10) $$\begin{array}{cccc} {\mathrm{Sa}}_{f}=0.1791E^{0.3614} & & & R^{2}=0.9999 \end{array}$$

(11) $$\begin{array}{cccc} {\mathrm{Sa}}_{w}=0.1359E^{0.3784} & & & R^{2}=0.9987 \end{array}$$

**Figure 11:**
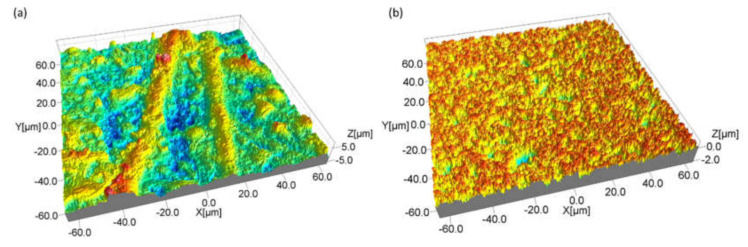
Details of machined surfaces for ZrB20f (**a**) and ZrB20w (**b**) machined by pulse type A.

**Figure 12:**
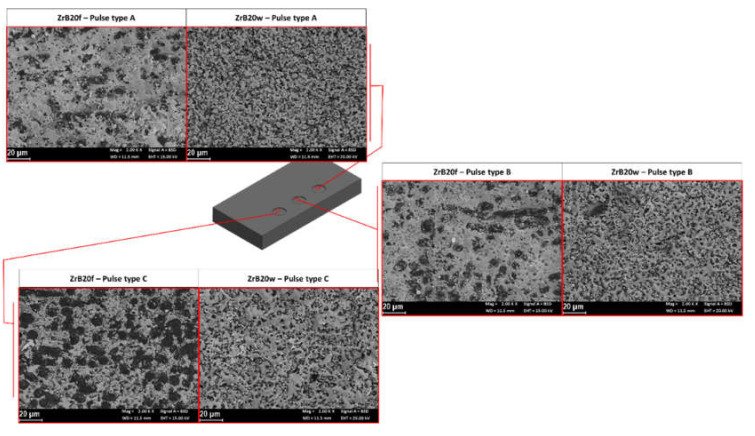
SEM backscatter images of the machined surface.

A 3D reconstruction of an ED-machined surfaces detail scanned by the confocal laser scanning microscope with a magnification of about 100× is reported as an example in Figure 11. In particular, Figure 11a represents a portion of the machined area on ZrB20f. Here it is possible to identify very clearly, a sort of “protrusion” in correspondence of the fibers. Such as reported in a previous work [20], this aspect is probably related to incomplete machining because of SiC low electrical conductivity characteristic and the great extension of the area of the fibers. Figure 11b represents the ED-machined surface for the sample containing SiC whiskers. In this case, the surface appears uniform and homogeneous because the SiC particles, such as reported in the materials section, are better dispersed in the base matrix. These aspects justified the different results obtained in terms of surface quality and, in general, these different textures can be considered a starting point for further studies about the material removal mechanism occurred on UHTCs, in particular when there is a low-electrically conductive parts in the structure. From the 3D reconstruction it is possible to observe that the surfaces are not characterized by the typical aspect of the ED-machined surfaces which present a texture well-described by the presence of craters. The different aspect has already been presented in a previous study [20]. In his specific case, the UHTC is different, but the same considerations can be done. A sort of craters can be observed by means of a SEM (Figure 12).

Machined surfaces on specimens doped with SiC whiskers are characterized by higher fragmentation of the recast layer. This aspect is particularly evident on surfaces machined by long pulses; in fact, for pulse type A and B, the surface is for the major part covered by recast materials for both additive shapes, but the specimens doped with whiskers presented smaller extensions of the single “crater” of recast materials.

Different behavior can be observed for surfaces machined by the short pulses. In this case, for specimens contained the SiC fibers can be observed a smaller area of recast material, probably because of the union of the greater dimensions of the fibers in comparison to the whiskers, the lower energy per discharge for short pulses and the low electrical conductibility of the SiC. In particular, the fibers have a bigger surface and it needs to work hard to remove the entire parts. For this reason, the surfaces contained fibers presented in their correspondence a sort of protrusion.

It is very clear that the whiskers affect in a significant way the micro-EDM process improving the energy efficiency and the machining performances increasing the machining speed and reducing the tool wear. Furthermore, the geometry and the behavior of whiskers during the samples preparation generate a homogenous surface improving the removal rate and reducing the risk of leave machining witness (as the fibers) on the surfaces which would generate better surface finishing. These considerations allow to suppose a different behavior of the discharges path as a function of the kind of material met and the distribution of the reinforcement.

## 5. Conclusions

An evaluation of the machinability of ZrB_2_-based composites hot-pressed with different shapes non-reactive additive (SiC) was performed in this work. Stability and repeatability of the micro-EDM were evaluated to identify the effects of the additive shapes. The analysis taking into account the process performances and surface finishing.

First of all, a discharge characterization was performed to feature the different pulse type used during the machining. In general, the discharge characterization and the performances indicators allowed to identify a stable and repeatable process with a faster material removal for samples doped with whiskers.

The analysis of variance showed that both factors, pulse type, and additive shape, are statistically significant for the indicators selected in the process evaluation and in general, the use of whiskers improve the material removal rate generating lower tool wear. Furthermore, the interaction between the two factors turns out to be influential only for MRD and TWD, which are indirectly related to the machining duration, since the number of discharges occurred during the machining was considered in their estimation.

This investigation shows that the specimens having a 20 vol.% of additive in form of whiskers results to be the best solution in terms of machinability by EDM process not only for the better process performances, but also for the higher level of surface quality which is one of the essential criteria for making a proper decision for industrial application.

## Figures and Tables

**Table 1:** Composition and sintering parameters of the hot-pressed samples [1].

Label	Composition	T_ON_ (°C)	T_MAX_ (°C)	Final Density (g/cm^3^)	Relative Density (%)
ZrB20f	ZrB_2_ + 20% SiC Short fibers	1545	1650	4.89	94.0
ZrB20w	ZrB_2_ + 20% SiC Whiskers	1560	1700	5.22	97.0

**Table 2:** Mean values of pulse type characteristics.

Additive Shape	Pulse Type	Peak Current (A)	Open Circuit Voltage (V)	Width (µs)	Energy per Discharge (µJ)
**Fibers** **(ZrB20f)**	**A**	29.44	69.57	0.70	779.36
	**B**	10.93	92.89	0.33	150.30
	**C**	4.61	100.79	0.06	14.73
**Whiskers** **(ZrB20w)**	**A**	29.49	70.75	0.70	784.99
	**B**	10.91	91.70	0.33	161.00
	**C**	4.10	99.60	0.06	21.41

**Table 3:** Results of the experimental campaign for process performances, where µ and σ identifies the average value and the standard deviation respectively.

Additive Shape	Pulse Type	$\mu_{{\mathrm{MRD}}_{\delta }}$	$\sigma_{{\mathrm{MRD}}_{\delta }}$	$\mu_{\mathrm{TWD}}$	$\sigma_{\mathrm{TWD}}$	$\mu_{{\mathrm{TWR}}_{\delta }}$	$\sigma_{{\mathrm{TWR}}_{\delta }}$	$\mu_{\mathrm{Sa}}$	$\sigma_{\mathrm{Sa}}$
		(µm^3^)	(µm^3^)	(µm^3^)	(µm^3^)			(µm)	(µm)
Fibers	A	61.40	1.19	44.22	0.65	0.720	0.009	1.997	0.152
	B	36.63	1.93	15.48	0.95	0.422	0.004	1.086	0.036
	C	5.91	0.09	1.29	0.05	0.218	0.005	0.475	0.011
Whiskers	A	63.82	2.10	40.54	0.20	0.636	0.019	1.720	0.178
	B	38.52	1.09	13.75	0.11	0.357	0.013	0.904	0.064
	C	12.18	0.20	1.60	0.02	0.132	0.004	0.439	0.019

**Table 4:** Analysis of variance *p*-values.

		FACTORS	
		Additive Shape	Pulse Type * Additive Shape
**INDICATORS**	**MRD_δ_ (µm^3^)**	1.21 × 10^−^^4^	0.03
	**TWD (µm^3^)**	0.00	4.51 × 10^−^^5^
	**TWR_δ_ [-]**	0.00	0.2019
	**Sa (µm)**	0.0045	0.156

* indicate the interaction between Pulse Type and Additive Shape
